# Hexavalent chromium removal from aqueous medium by ternary nanoadsorbent: A study of kinetics, equilibrium, and thermodynamic mechanism

**DOI:** 10.1371/journal.pone.0290234

**Published:** 2023-12-22

**Authors:** Md Nashir Uddin, Ganesh Chandra Saha, Md Abul Hasanath, M. A. H. Badsha, Mohaiminul Haider Chowdhury, Abu Reza Md. Towfiqul Islam

**Affiliations:** 1 Department of Civil Engineering, Dhaka University of Engineering and Technology, Gazipur, Bangladesh; 2 Department of Civil Engineering, Indian Institute of Technology, Hyderabad, India; 3 Department of Civil and Environmental Engineering, California Polytechnic State University, San Luis Obispo, CA, United States of America; 4 Institute of Water and Environment, Dhaka University of Engineering & Technology, Gazipur, Bangladesh; 5 Department of Disaster Management, Begum Rokeya University, Rangpur, Bangladesh; 6 Department of Development Studies, Daffodil International University, Dhaka, Bangladesh; AGH Faculty of Mining Surveying and Environmental Engineering: Akademia Gorniczo-Hutnicza im Stanislawa Staszica w Krakowie Wydzial Geodezji Gorniczej i Inzynierii Srodowiska, POLAND

## Abstract

Although many studies have focused on chromium removal from aqueous media by ternary Nano adsorbents, still the integrated kinetics, equilibrium, and thermodynamic mechanisms of chromium removal remain unknown. Thus in this study, we have synthesized a novel ternary oxide nanocomposite comprising iron, manganese, and stannous (Fe_2_O_3_-MnO_2_-SnO_2_) in a facile method as a promising adsorbent for the removal of Cr(VI) from an aqueous medium. The Fe_2_O_3_-MnO_2_-SnO_2_ system was firstly characterized by FTIR, XRD, TGA, BET, and SEM/EDX. The effect of parameters, for instance, pH, temperature, initial Cr(VI) intensity, and adsorbent dose, have been examined to optimize the Cr(VI) adsorption performance. The adsorption of Cr(VI) onto Fe_2_O_3_-MnO_2_-SnO_2_ nanoadsorbent is associated with an adsorption/reduction mechanism. Using an initial Cr(VI) intensity of 50 mg L^-1^, 200 rpm agitation, 2.5-g L^-1^ of adsorbent, pH 2, 90 minutes adsorption time, and 298 K temperature, a maximum adsorption capability of 69.2 mg Cr(VI) g^-1^ for Fe_2_O_3_-MnO_2_-SnO_2_ was obtained. Models of pseudo-2^nd^-order kinetics and Langmuir’s isotherm were best suited to the investigated data. Besides, thermodynamic parameters show that Cr(VI) adsorption onto Fe_2_O_3_-MnO_2_-SnO_2_ was random and dominated by entropy. The reusability of Fe_2_O_3_-MnO_2_-SnO_2_ was found to be consistently high (remaining above 80% for Cr(VI)) over four adsorption-desorption cycles. Chromium adsorption from the tannery wastewater was achieved 91.89% on Fe_2_O_3_-MnO_2_-SnO_2._ Therefore, Fe_2_O_3_-MnO_2_-SnO_2_ nanoparticles, being easy to be synthesized, reusable and having improved adsorption capability with higher surface area, could be a desirable option for removing Cr(VI) from aqueous environments.

## Introduction

The accelerated industrialization and urbanization in recent decades have resulted in frequent introduction of numerous pollutants into the natural setting. This influx of pollutants has contributed to an increased local environmental quality deterioration, which disrupts the stability of the surrounding ecosystem and threatens individuals [1]. Instances of heavy metal pollution are a recurring phenomenon on a global scale, resulting in significant contamination of aquatic ecosystems. Water pollution caused by heavy metal ions released into aquatic systems by industrial waste, one of the most prominent contributors to environmental contaminants, is a critical environmental issue owing to its excessive noxiousness and bio-accumulative aspect. Heavy metals such as arsenic, copper, mercury, chromium, zinc, lead, and nickel are known to possess ionic species that have been identified as carcinogenic to humans [2]. These metals are released actively or indirectly into the environment through wastewater from tanneries, electroplating industries, mining operations, and paper mills [3]. Chromium is enumerated as carcinogenic among the 50 most harmful metals on Earth by the World Health Organization. The discharge of hexavalent chromium into the surrounding ecosystem has been documented as a consequence of various industrial operations, such as tanning, metal plating, dyeing, and manufacturing paints and pigments, among other activities [4]. Because of extensive industrial and manufacturing applications, chromium is often identified in surface water and groundwater exceeding 0.1 mg L^-1^.

The maximum tolerable concentration of Cr(VI) in surface water from industrial effluents is 0.1 mg L^-1^, whereas in drinking water it’s 0.05 mg L^-1^ [5]. In the aqueous phase, chromium is mainly found with valency three and six species, denoted as (Cr(III)) and (Cr(VI)), respectively, whereas hexavalent species of chromium, e.g., chromate (CrO_4_^2−^, HCrO_4_^−^) and dichromate (Cr_2_O_7_^2−^) are more harmful to humans than trivalent species [6]. They are highly soluble and reactive in aqueous media. Additionally, Cr(VI) has mutagenic, carcinogenic, genotoxic, and teratogenic consequences on living beings [7]. Chromium must, therefore, be removed from industrial effluents as a prime concern to protect human and environmental health.

There are various physical and chemical methods to remove Cr(VI) from water/wastewater: precipitation, coagulation (including redox-aided coagulation), ozonation, ultrafiltration, reverse osmosis, electrodialysis, ion exchange, and membrane technology, etc. [8]. However, despite being highly effective in removing aqueous Cr(VI), each method has limitations, e.g., high costs and operating challenges [9]. Adsorption may be a superior option for removing toxic metals because of the simplicity of operation and relatively high metal extraction effectiveness [10]. As such, numerous adsorbents, for instance, activated carbon [11], starch stabilized hybrid nanomaterials, stannous-based nanoparticles, chitosan, *α*-Fe_2_O_3_/*γ*-Fe_2_O_3_/Fe/C nanocomposite, decorated polypyrrole magnetic composite of reduced graphene oxide-Fe_3_O_4_, and manganese-based nanoparticles, were tested to remove Cr(VI) from aqueous media [12].

Numerous techniques have been used to develop nanoadsorbent materials, such as co-precipitation, ion beam deposition, sol-gel, microemulsion, colloidal method, spray pyrolysis, template synthesis, laser pulse, ball milling, biological synthesis, as well as chemical vapor deposition [13]. Researchers find it very hard to make adsorbents with a high adsorption capacity (mg g^-1^) and excellent specificity toward target contaminants [14].

Iron oxide being highly effective in removing various pollutants, interest in using iron oxide nanotechnology to address environmental issues among the researcher has been seen in a spiking trend. Iron oxide nanoparticles possess a significantly greater surface area and a higher number of vacant sites, rendering them highly suitable for functioning as an ideal adsorbent and that’s why researchers are using iron-based nanocomposites for water treatment [15]. Besides, iron is considered to be a suitable and environmentally advantageous substance for the purpose of treating effluent due to its cost-effectiveness, benign nature, and convenient fabrication process. Magnetite (Fe_3_O_4_), maghemite (γ-Fe_2_O_3_), hematite (α-Fe_2_O_3_), iron oxy-hydroxide (FeOOH), and metallic zero-valent iron represent a range of iron-based nanomaterials. Researchers have previously developed and tested iron oxide-based nanoparticles for Cr(VI) removal [16–18] Hu et al. studied the performance and mechanism of chromate (VI) adsorption by FeOOH-coated Fe_2_O_3_ nanoparticles using sol-gel method and found the adsorption capacity 25.8 mg g^-1^ at pH 2.5 [16]. Wei et al. reported iron–nickel oxide as an effective adsorbent that was prepared by the coprecipitation method and maximum adsorption capacity was reported 30 mg g^-1^ for Cr(VI) ions [17]. Besides, Yuan et al. studied diatomite-supported/unsupported magnetite nanoparticles with maximum adsorption capacity of 15.3 mg g^-1^ for Cr(VI) [18]. Also, the utilization of Sn(II) oxides has demonstrated considerable potential as an effective materials in addressing removal of Cr(VI) in potable water treatment as Sn(II) can provide two electrons (Sn^2+^—>Sn^4+^ + 2e^−^) for Cr(VI) reduction. Furthermore, considering the low toxicity of tin and the extremely low solubility of tin compounds, the potential existence of dissolved tin in drinking water is not regarded as a significant risk to human health [19]. Numerous experiments have demonstrated the performance of tin for Cr(VI) removal from the water/wastewater [19, 20]. Most of the researcher applied Sn-based nanoparticle for the photocatalytic reduction of aqueous Cr(VI) under visible light. Bayat et al. reported maximum adsorption capacity 53.52 mg g^-1^ at acidic environment of pH 5.0 [19]. On the other hand, Kaprara et al. observed maximum uptake capacity of around 19 mg g^-1^ utilizing Sn-based nanoadsorbent in high acidic environment (pH 2.0) [20]. Besides, manganese oxide-based nanoparticles also showed their capability as a prospective adsorbents for Cr(VI) removal from the aqueous media [21–23]. Luther et al. reported Cr(VI) binding to manganese and Iron oxide nanoparticles under light and dark conditions [23]. They found that maximum capacity of Cr(VI) uptake under light and dark condition are 3.21 mg g^-1^ and 3.87 mg g^-1^, respectively. Cantu et al. utilized manganese oxide nanomaterials in the adsorption of Cr(VI) and reported maximum uptake capacity 2.5 mg g^-1^, 4.3 mg g^-1^, and 5.8 mg g-1 for 4°C, 21°C, 45°C, respectively [21]. N. Li et al. studied Cr(VI) adsorption onto magnetic mesoporous MnFe_2_O_4_@SiO_2_-CTAB composites and stated maximum 25.044 mg g^-1^ of Cr(VI) has been adsorbed by the synthesized nanomaterials [22]. Therefore, synthesis of Fe, Mn, and Sn ternary oxides is possible as they have potent oxidation properties (from the manganese dioxide), excellent adsorption properties (from the iron and stannous oxides), and superior separation properties.

The use of ternary phased NCs as adsorbents to remove a wide variety of organic or inorganic contaminants has expanded over the past several years. Differences between ternary adsorbents and other adsorbents may be described to several distinguishing attributes like ease of fabrication, a large surface ratio to volume, rapid performance during the adsorption process, the ability to readily separate and regenerate, and so on [24]. T. Wang et al. created a ternary nanoparticle with the composition of Fe_3_O_4_/g-C_3_N_4_/CL using a one-step hydrothermal carbonization approach to remove Cr(VI) [25]. Li et al. proposed a mechanochemical technique for producing a ternary nanocomposite comprising polyethyleneimine (PEI), magnetite (Fe_3_O_4_) nanoparticles, and illite/smectite clay nanoflakes using a high-energy density stirred bead mill [26]. Chen et al. developed ZnTiO_3_/Zn_2_Ti_3_O_8_/ZnO ternary heterostructure by solvothermal-calcination method [27]. Chachvalvutikul et al. synthesized dual Z-plan Ternary heterojunction photocatalyst of FeVO_4_/Bi_4_O_5_Br_2_/BiOBr for the simultaneous photocatalytic elimination of Cr(VI) [28]. Typically, two or more chemical processes are involved in the creation of ternary composites that are costly and time-consuming. Sol-gel method has some advantages over other methods, including low operating temperatures and the capacity to produce complex structures and composite materials and the technique would be both cost-effective and environmentally friendly. Considering the wide range of benefits, the sol-gel technique has been employed in this study. However, to the best of our knowledge, the synthesis and application of ternary nanoparticles (formed by combining iron, manganese, and stannous) for Cr(VI) removal have not yet been studied. With a focus on removing Cr(VI) from aqueous media, to fill the research gap, this research sought to create a ternary novel nanoparticle with enhanced sorption properties such as surface area, sorption capacity, and reusability. Accordingly, in this work, a novel ternary Fe_2_O_3_-MnO_2_-SnO_2_ nano adsorbent was prepared by the Sol-gel method and characterized by XRD, FTIR, TGA, BET, SEM, and EDX analysis. The adsorption performance and stability of a ternary nano-oxide adsorbent composed of Fe_2_O_3_, MnO_2_, and SnO_2_ were evaluated in Cr(VI) adsorption by looking at how several factors, including initial metal concentration, adsorbent dose, contact time, agitation, and temperature, influenced the rate of adsorption. Experiments were conducted to know the adsorption isotherm and kinetics and to evaluate the efficacy of regenerating and reusing the nanoadsorbent. Fe_2_O_3_-MnO_2_-SnO_2_ NPs’ demonstrated promising results in removing Cr(VI) from aqueous media.

## Materials and method

### Chemicals

Analytical grade tin (II) chloride (SnCl_2_.2H_2_O), potassium permanganate (KMnO_4_), iron chloride (FeCl_2_.6H_2_O), sodium hydroxide (NaOH), and potassium dichromate salts (K_2_Cr_2_O_7_), were used in the study. All the chemicals utilized in this study were manufactured by Sharlab S L Spain and no refinement was made to those during preparing the solutions with deionized (DI) water.

### Synthesis of Fe_2_O_3_-MnO_2_-SnO_2_ nanoparticles

**Fig 1:**
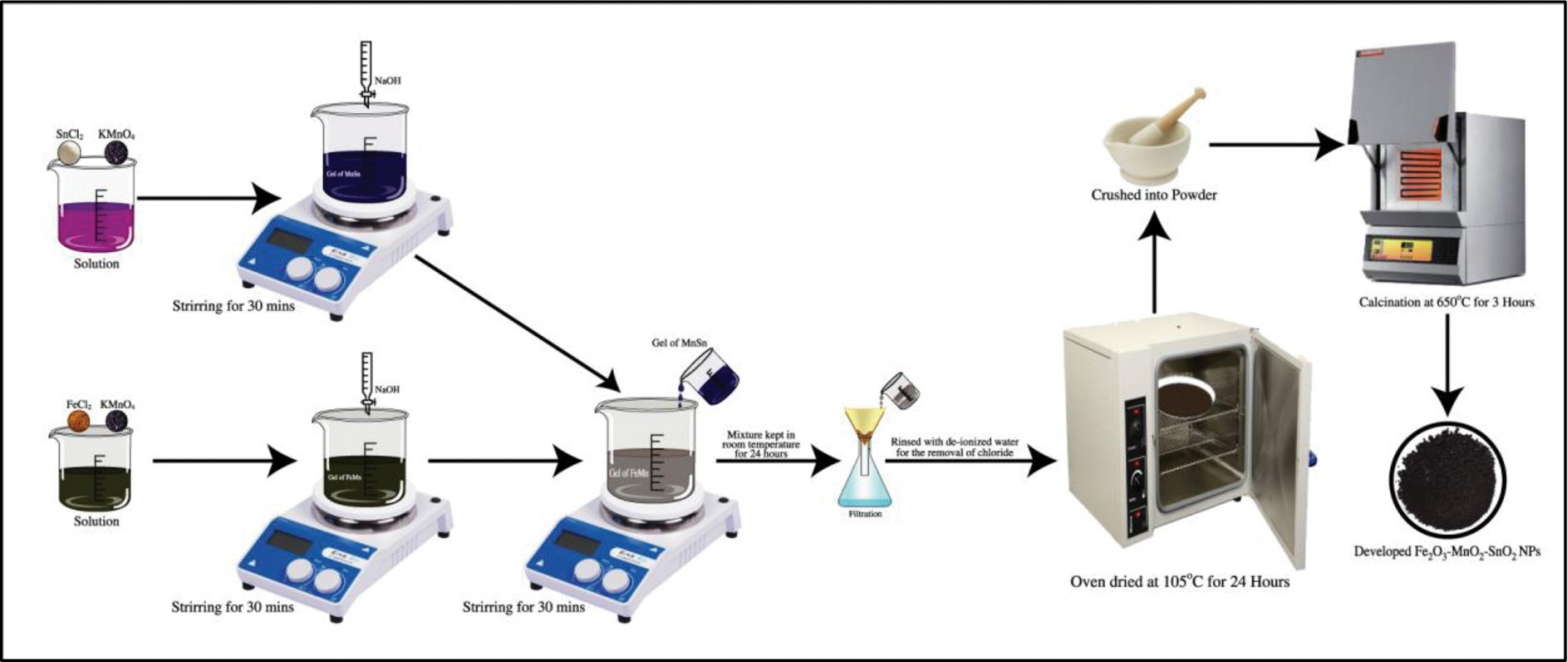
Graphical representation of synthesis of Fe_2_O_3_-MnO_2_-SnO_2_ nanoparticles.

This research used the sol-gel method to prepare the ternary Fe_2_O_3_-MnO_2_-SnO_2_ nanoadsorbent [29]. The procedure is as follows: “First, potassium permanganate (KMnO_4_) and iron chloride (FeCl_2_) were dissolved separately in 100 mL of deionized water. The KMnO_4_ solution was then gradually mixed into the FeCl_2_ solution under dynamic magnetic stirring for 30 minutes to resulting the decreasing of the solution pH to a range of 3‒4. According to Hu et al. Fe-based adsorbent synthesis maintaining pH around 8.0 provide good adsorbent properties [16] and so, to maintain the pH of the solution at 8, and developing gels of manganese and iron hydroxide, a 2M NaOH solution was added drop-wise into the mixture. The stirring continued for 30 minutes. A hydroxide gel of tin and manganese was also prepared following similar procedure. This gel was then added dropwise to the iron and manganese gel under magnetic stirring for 1 hour. A gel-like precipitate of hydroxide was thus developed and was then put in the mother liquor for 24 hours’ [29]. The solution was then allowed to stabilize at room temperature for 24 hours and then it was filtrated using Whatman filter papers (cat no 1001 110). The product is then accumulated as a precipitate and rinsed with deionized (DI) water for 12 times to get rid of unbound chemicals. Finally, the solid precipitate was oven-dried at 105°C, pulverized into a powder, followed by 3 hours of calcination at 650°C to have the newly developed Fe_2_O_3_-MnO_2_-SnO_2_ nanoparticles. **Fig 1** depicts the graphical representation of the synthesis procedure.

### Batch studies

Batch studies were conducted to measure the Fe_2_O_3_-MnO_2_-SnO_2_ nanomaterial’s ability to adsorb Cr(VI) ions, where the Cr(VI) stock solutions were formulated with 1000 mg L^-1^ of concentration by mixing 2.84 g of 99% K_2_Cr_2_O_7_ with deionized water in a 1000 mL flask. The stock solution was diluted where necessary while preparing the samples. The pH was adjusted by means of 0.1 M of NaOH or HCl. Unless otherwise specified, the adsorption test was carried out by introducing 2.5 g L^-1^ of Fe_2_O_3_-MnO_2_-SnO_2_ nanoadsorbent at room temperature (25°C) by agitating it at a frequency of 200 rpm in a 250 mL glass bottle consisting of 100 mg L^-1^ Cr(VI) solution. After 2 hours of agitation (aside from adsorption kinetics), the suspension was filtered and the residual concentration of Cr(VI)was measured by Shimadzu AA-7000 Atomic Absorption Spectrophotometer (AAS). The removal efficiency, adsorption capability at equilibrium, and adsorption capability at time *t* were determined by Eqs 1–3 provided in the supplementary document.

$$\%Removal=\frac{C_{o}-C_{e}}{C_{o}}x100$$ (1)

$$q_{e}=\frac{C_{o}-C_{e}}{m}xV$$ (2)

$$q_{t}=\frac{C_{o}-C_{t}}{m}xV$$ (3)

where *C*_*o*_ and *C*_*e*_ are the initial and equilibrium concentrations of Cr(VI), respectively, measured in mg L^-1^. The adsorption capacity *q*_*e*_ and *q*_*t*_ are the quantities of Cr(VI) adsorbed per unit weight of Fe_2_O_3_-MnO_2_-SnO_2_ nanoadsorbent at equilibrium and at a specific time *t* (mg g^-1^) individually, where % removal is the efficiency of Cr(VI) removal, *m* is the mass of adsorbent applied (g), and *V* is the volume of the Cr(VI) solution (*L*).

To identify the effect of pH on adsorption was observed with 100 mg L^-1^ of Cr(VI) solution after varying the solution’s pH from 1 to 9 via 0.01 M NaOH/HCl. Other parameters were held constant: 2.5 g L^-1^ adsorbent dosing, 60 minutes contact time, 25°C temperature and agitation was at 200 rpm. Besides, the effect of Fe_2_O_3_-MnO_2_-SnO_2_ ternary nanoadsorbent dosage was studied under 0.5, 1.0, 1.5, 2.0, 2.5, 3.0, 5.0, 10.0, 15.0, 20.0, 25.0, and 30.0 g L^-1^ while the other parameters were: pH 2.0, contact time 90 minutes, agitation speed 200 rpm, initial Cr(VI) concentration 100 mg L^-1^, and temperature 25°C. Moreover, the effect of initial Cr(VI) ion concentration on adsorption was decided with a series of Cr(VI) ion solution containing concentrations: 5 to 300 mg L^-1^, keeping all other variables constant as same as nanoadsorbent dosage studies.

### Adsorption isotherm

The adsorption isotherm study is significant as it expresses how adsorbates are distributed in the solid phase once they reach equilibrium. It is also a crucial indicator of a specific adsorbent material’s effectiveness. Isotherm data usually fits one of two models: the Langmuir model, or the Freundlich model. If the experimented data suitably matches the Langmuir isotherm model, that implies the adsorbent’s surface is homogeneous and adsorption is achieved via monolayer sorption on the surface [30]. The non-linear adsorption isotherm models devised by Langmuir and Freundlich were employed to fit the experimental data to determine the maximum Cr(VI) adsorption capacity of the Fe_2_O_3_-MnO_2_-SnO_2_.

In the adsorption isotherm study, the initial Cr(VI) concentration, adsorbent dosage, and temperature were considered in the range of 50−300 mg L^-1^, 2.5-g L^-1^, and 25°C, respectively. Eq 4 express the Langmuir isotherm model in non-linear forms [31].

$$q_{e}=\frac{q_{m}K_{L}C_{e}}{1+K_{L}C_{e}}$$ (4)

where *q*_*e*_ is a sorbent’s equilibrium adsorption capability (mg g ^-1^), *C*_*e*_ is the equilibrium intensification of the sorbate ion (mg L^-1^), *q*_*m*_ is the optimum potential for adsorbing a metal monolayer for a particular adsorbent in mg g^-1^, and *K*_*L*_ denotes the constant which corresponds to the adsorption bonding energy in L mg^-1^.

To evaluate the values of *q*_*m*_ and *K*_*L*_, non-linear regression analysis was carried out using the software Origin (OriginLab Corporation). Also, the Langmuir isotherm’s crucial phenomenon, a nondimensional constant (*R*_*L*_) that shows whether adsorption is favorable or not was determined by Eq 5.

$$R_{L}=\frac{1}{1+K_{L}C_{0}}$$ (5)

where *C*_*0*_ is the initial concentration of Cr(VI) (mg L^-1^) and *K*_*L*_ is the Langmuir concentration (L mg^-1^). *R*_*L*_, which specifies the favorability of the adsorption process, may be used to determine the isotherm pattern. This could be (i) *R*_*L*_ = 0, (ii) *R*_*L*_ = 1, (iii) *R*_*L*_ > 1, or (iv) 0 > *R*_*L*_ < 1, to respectively denote the cases where adsorption is irreversible, linear, unfavorable, or favorable.

If, in contrast, the data fits the Freundlich isothermal model, then that clearly indicates the adsorbent’s surface is heterogeneous and adsorption is achieved via multilayer sorption, as expressed in Eq 7 [31].

$$q_{e}{=K}_{F}C_{e}^{\frac{1}{n}}$$ (6)

where *C*_*e*_ is the Cr(VI) ions (mg L^-1^) concentration at equilibrium, *q*_*e*_ is the amount of Cr(VI) adsorbed at equilibrium (mg g^-1^), *K*_*F*_ is the constant of Freundlich isotherm concerning adsorbent capacity, and *n* is the experimental constant about adsorbent extent divergent from the adsorbent’s heterogeneity. The adsorbent’s surface possessions and affinity are characterized by certain consistent values that can also be applied to contrast the uptake efficiency of metal [32].

The Temkin model takes into account the implications of adsorbate-adsorbate interactions. It is assumed that all molecules in the layer exhibit a linear decrease in heat of adsorption with increasing surface coverage, as opposed to a logarithmic decrease. The Temkin isotherm was expressed using the following nonlinear form Eq 7:

$$q_{m}=\frac{RT}{B_{T}}ln(K_{T}C_{e})$$ (7)

In Eq (7), R represents the gas constant with a value of 8.314 J mol^-1^ K^-1^, T denotes the absolute temperature measured in Kelvin. K_T_ signifies the Temkin isotherm equilibrium binding constant expressed in units of L g^-1^. Lastly, B_T_ represents the Temkin isotherm constant associated with the heat of sorption, measured in J mol^-1^.

### Adsorption kinetics

To determine the adsorption kinetics, 2.5 mg L^-1^ of the adsorbent was dosed into 100 mL of a solution containing 180 mg L^-1^ of Cr(VI). Samples were collected at different time intervals (0–300 minutes) and their residual Cr(VI) concentrations were measured to calculate the Cr removal efficiency. Common kinetics models, pseudo-first-order (Eq 8), pseudo-second-order (Eq 9), Elovich (Eq 10) were used to fit the empirical data [33].

$$q_{t}=q_{e}[1-exp(-k_{1}t)]$$ (8)

$$q_{t}=\frac{q_{e}^{2}k_{2}t}{{1+q_{e}k}_{2}t}$$ (9)

$$q_{t}=\frac{1}{\beta }ln(1+\alpha \beta t)$$ (10)

where *q*_*e*_ (mg g^-1^) and *q*_*t*_ (mg g^-1^) refer to the sums of Cr(VI) adsorbed by Fe_2_O_3_-MnO_2_-SnO_2_ at equilibrium and time *t*, sequentially; the rate constants for pseudo-first- and second-order kinetics are *k*_*1*_ (min^-1^) and *k*_*2*_ (g mg^-1^ min^-1^), respectively. Besides, α (mg g^-1^ min^-1^) is the initial adsorption rate, β (g mg-1) is the desorption constant related to the extent of surface coverage and activation energy for chemisorption. The identification of diffusion mechanisms is not possible based on the aforementioned kinetic models. The explanation of the intra-particle diffusion model can be found in Eq 11 [34] as presented below:

$$q_{t}=K_{id}t^{1/2}+C$$ (11)

The intraparticle diffusion rate constant (K_id_) is denoted in units of mg g^-1^ min^-1/2^, while C represents the intercept.

### Influence of temperature and thermodynamic analysis

100 mg L^-1^ of Cr(VI) was used in the batch experiment to test the influence of temperature on Cr(VI) adsorption. Other experimental settings, such as pH, adsorbent dosage, and contact time were kept constant. The thermodynamic parameters are performed in the range of 298‒313 K to reveal the Fe_2_O_3_-MnO_2_-SnO_2_’s Cr(VI) ion adsorption characteristics. These parameters are associated with standard Gibbs free energy (ΔG°), standard enthalpy (ΔH°), and entropy (ΔS°) [35]. The equilibrium constant and the transition in Gibbs free energy were computed using Eqs 12 and 13. Therefore, ΔH° and ΔS° were calculated from the Van’t Hoff formula (Eq 14) derived from a linear method [36].

$$\Delta G{}^{\circ}=-RTlnK_{c}$$ (12)

$$K_{c}=q_{e}/C_{e}$$ (13)

$$lnK_{c}=\frac{{\Delta S}^{{}^{\circ}}}{R}-\frac{{\Delta H}^{{}^{\circ}}}{R.T.}$$ (14)

where *K*_*c*_ is the thermodynamic constant at equilibrium, *q*_*e*_ (mg g^-1^) is the quantity of Cr(VI) adsorbed, *C*_*e*_ (mg L^-1^) is the Cr(VI) density in the solution at equilibrium, and *R* denotes the ideal gas constant while *T* denotes temperature (K) [35].

### Desorption and regeneration study

Desorption of Cr(VI) was performed firstly in distilled water, followed by a basic medium and an acidic medium, to identify a suitable eluant for Cr(VI). To regenerate the adsorbents, adsorbed Cr(VI) from exhausted nanoadsorbent was experimented using three different solvents (HCl, HNO_3_, and NaOH) at these molar ratios: (i) 0.10, 0.50 and 1.0-M HCl; (ii) 0.10, 0.50, and 1.0 M HNO_3_; and (iii) 0.10, 0.50, and 1.0 M NaOH.

## Results and discussions

### Structural characteristics of Fe_2_O_3_-MnO_2_-SnO_2_ ternary nanoadsorbent

#### FTIR analysis

**Fig 2:**
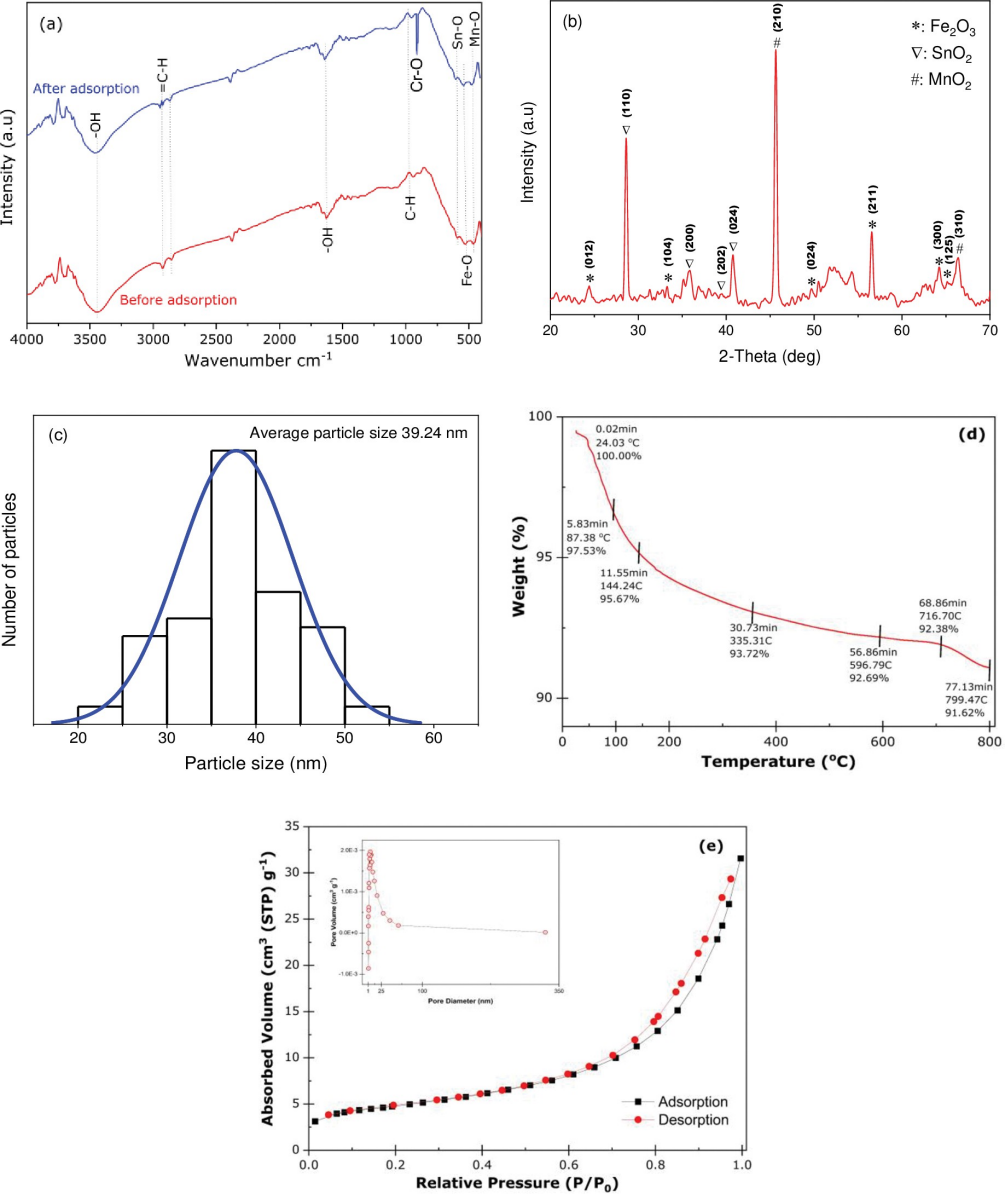
(a) FTIR spectrum, (b) XRD pattern, and (c) size distribution histogram of synthesized Fe_2_O_3_-MnO_2_-SnO_2_ nanoparticle (d) TGA Thermogram of the Fe_2_O_3_-MnO_2_-SnO_2_ nanoparticle, (e) Nitrogen adsorption–desorption isotherm (inset) pore size of the Fe_2_O_3_-MnO_2_-SnO_2_ nanoparticle.

The Fourier Transform Infrared (FTIR) spectrum (band between 400 and 4000 cm^-1^) has been used to define the functional groups of the developed Fe_2_O_3_-MnO_2_-SnO_2_ nanoadsorbent. Fig 2(A) shows that the bands found at 1627 cm^-1^ and 3452 cm^-1^ result from the bending oscillation of the absorbed water and OH^-^ stretching, respectively. Further, the sharp bands between 3000–2850 cm^-1^ and 1000‒650 cm^-1^ are attributable to the = C-H and C-H bonds [37]. The absorbance peaks in the fingerprint region, beneath wavelength 1000 cm^-1^, mostly originate from interatomic vibrations due to the presence of hydroxides and oxides in the structure of the nanoparticles. The sharp band at 590 cm^-1^ is attributed to the Sn-O bond of SnO_2_ [38], while the band at 528 cm^-1^ belongs to the Fe-O vibration [39]. Also, the band detected at 466 cm^-1^ is ascribed to the O-Mn-O bond [40]. The existence of the respective bands in the FTIR data, which pertain to the different functional groups in Fe_2_O_3_-MnO_2_-SnO_2_, confirms the formation of the ternary nanoadsorbent without impurity. A small shift in the characteristic peak of the IR absorption spectrum was observed after Cr(VI) adsorption when comparing the spectra before and after adsorption to the Fe_2_O_3_-MnO_2_-SnO_2_ adsorbent. The stretching vibration of the O-H bond in the absorption peak shifted from 3452 cm^-1^ to 3449.225 cm^-1^ and 1627 cm^-1^ to 1643.35 cm^-1^, indicating to the participation of both O-H stretching and H-O-H bending vibrations in the adsorption. A new peak appears at 912.40 cm^-1^ when comparing spectra before and after adsorption. These spectral shifts are evidence that chromium has been bound to the adsorbents.

#### X-ray diffraction analysis of Fe_2_O_3_-MnO_2_-SnO_2_ nanoadsorbent

The crystal structure of the Fe_2_O_3_-MnO_2_-SnO_2_ nanoparticles was authenticated via peaks detected by X-ray diffraction (XRD). Fig 2(B) presents the characteristic peaks of the Fe_2_O_3_-MnO_2_-SnO_2_ nanoparticles. Patterns in XRD ranging between 20° and 70° are reported in 2θ. Miller indices are used in the identification of crystallographic planes. The XRD patterns of pure Fe_2_O_3_ were identified with the crystal planes (012), (104), (024), (211), (300), and (125), at 2θ of 24.40°, 33.46°, 49.58°, 56.54°, 64.19°, and 56.4°, respectively. The peak sample position reveals the rhombohedral structure of Fe_2_O_3_ (maghemite), indexed by the JCPDS Card No 84–0310 [41]. The 28.68° (110), 35.85° (200), 39.92° (202), and 45.62° (024) diffraction peaks all correspond to the SnO_2_ tetragonal form, indexed by JCPDS Card No 77–2296 [42]. The distinctive peaks are attributed to the tetragonal MnO_2_ form, indexed by JCPDS Cards No. 81–2261, at 40.67°, 45.87°, and 66.98°, corresponding to the (200), (210) and (310) planes, respectively [43]. The intense diffraction rate shows that the specimens are strongly crystalline upon the 3-hour calcination at 650°C [44]. The crystalline size D is appraised by applying the formula given by Debye-Scherrer [45]. The XRD data has shown the crystalline particle size to vary between 20.52 nm to 54.99 nm (histogram graphics distribution in Fig 2(C)) and averages to 39.27 nm.

#### Thermal gravimetric analysis (TGA)

The thermal stability of the Fe_2_O_3_-MnO_2_-SnO_2_ nanoparticle was evaluated using TGA, as shown in Fig 2(D), throughout a temperature range of 20°C to 800°C. According to the thermogram, the nanocomposite passed through six mass degradation phases, resulting in a weight reduction of 8.38 wt%. The First stage was recognized at 24.03–87.38°C where the weight reduction has nee observed 2.47%. The second to fourth stage has been observed at 87.38–144.24, 144.24–335.31 and 335.31–596.79°C respectively with 6.28% weight loss which is it may evaporate water that has been adsorbed to the surfaces of this nanoparticle [46]. The temperature range of 596.79–716.70°C was found corresponding to the fifth reduction step and above 799.47°C, the final step of thermal degradation was seen. At this temperature, the sample showed a minuscule weight loss of only 1.07%, which indicated that the nanoparticle had finally been transformed into carbon and the stable oxides of iron, stannous and manganese [46]. The total weight loss is 8.38%, which provides a good indicator of the formation of the nanocomposite and identifies no other impurity on the nanoparticles’ surface.

#### Surface area analysis

Brunauer–Emmett–Teller (BET) isotherm technique was undertaken to assess the surface area and porousness of the developed Fe_2_O_3_−MnO_2_−SnO_2_. In addition to this, pore size distribution of the developed nanoparticle was evaluated by Barrett–Joyner–Halenda (BJH) approach. Isotherms of the nanoparticle’s adsorption-desorption of nitrogen are portrayed in Fig 2(E). According to the BET isotherm curve, the nanocomposite demonstrated characteristics of a type IV isotherm in its behavior, indicating it is a mesoporous substance [47]. The total pore volume and surface area (P/P_0_ 0.990) of the Fe_2_O_3_-MnO_2_-SnO_2_ that was figured out via the BET technique and assessed at standard temperature and pressure (STP) was found 4.6015 x 10^−2^ cm^3^ g^-1^ and 14.539 m^2^ g^-1^ respectively. The Barrett–Joyner–Halenda (BJH) approach found that the nanoparticle possesses a mean pore diameter of 4.96nm. This number provides evidence that the nanoparticle formation is mesoporous and that the pores are all of identical dimensions [48]. With more active important sites for adsorbing metal ions like Cr(VI), this characteristic increases the adsorptive extraction efficiency.

#### Morphology analysis of Fe_2_O_3_-MnO_2_-SnO_2_

**Fig 3:**
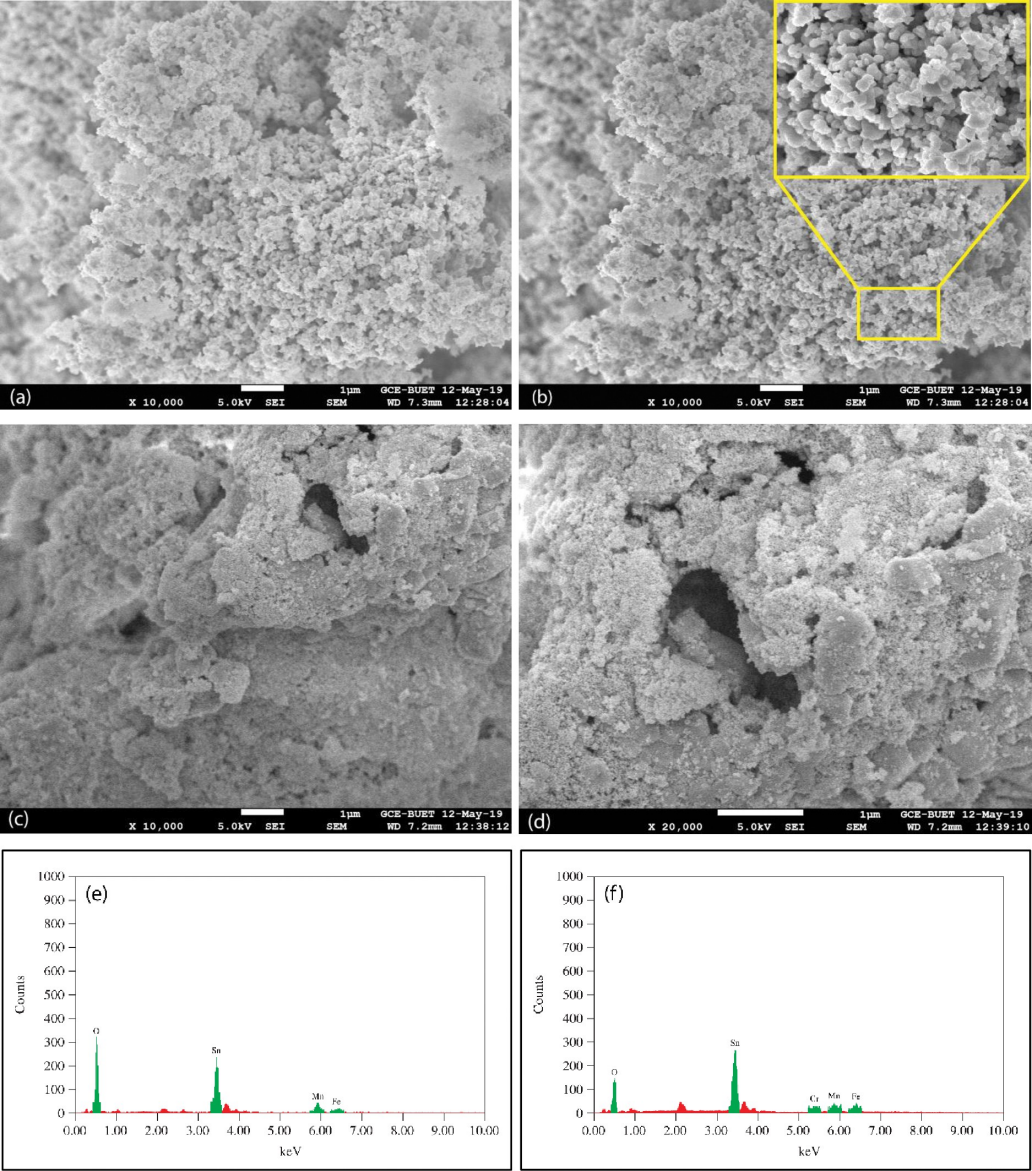
SEM image of Fe_2_O_3_-MnO_2_-SnO_2_ NPs (a–c) before Cr(VI) adsorption, (d–f) after Cr(VI) adsorption, (g) EDX spectrum before Cr(VI) adsorption, and (h) EDX spectrum after Cr(VI) adsorption.

The morphology of Fe_2_O_3_-MnO_2_-SnO_2_ nanoadsorbent was characterized by SEM micrographs and the scanning images exposed several key structural attributes in Fig 3(A) and 3(B). These images show that several large and small aggregated particles have formed within the materials and their surfaces vary from heterogeneous, irregular, rough, to porous textures. The results show that several distinctly formed and sized pores possess profound and highly interconnected, infrequent large areas that exist on the surface of the Fe_2_O_3_-MnO_2_-SnO_2_ nanoparticles. This loose arrangement and surface pores support molecular mobility and allow the molecules enough space to adsorb Cr(VI) from an aqueous medium. The Fe_2_O_3_-MnO_2_-SnO_2_ nanoparticles’ surface is porous, resulting in greater surface area and pore volume. The porosity of the adsorbent material is crucial for higher adsorption qualities in water environments since the higher the surface area, the higher the adsorption capacity [49]. However, after the adsorption of Cr(VI) onto the Fe_2_O_3_-MnO_2_-SnO_2_ nanoadsorbent, the particles’ topography turns rough (Fig 3(C)–3(E)). Furthermore, honeycomb-like pores have been found in the Cr(VI)-adsorbed samples (Fig 3(C) and 3(D)). Transformations in surface conditions of the particles confirm that they bind with Cr(VI) ions during adsorption.

The chemical compositions of the Fe_2_O_3_-MnO_2_-SnO_2_ nanoparticles were determined employing EDX in the SEM. Fig 3(E) illustrates the presence of Fe, Sn, Mn, and O in the specimen. The peaks at 6.40, 5.9, 3.44, and 0.52 keV correspond to the binding energy of Fe, Mn, Sn and O, respectively. The EDX study shows Fe, Mn, Sn, and O to be the fundamental constituents spread on every part of the surface of the Fe_2_O_3_-MnO_2_-SnO_2_ with respective weight percentages of 2.97, 5.24, 3.04, and 88.75 (Table 1). After the adsorption of Cr(VI), an analysis was conducted using EDX in the SEM to know the elemental composition. Fig 3(F) illustrates that the existence of Cr with Fe, Mn, Sn, and O in the sample and it evidences that Cr(VI) was, indeed, adsorbed onto the Fe_2_O_3_-MnO_2_-SnO_2_. Besides, Table 1 represent the existence of Fe, Mn, Sn and O along with adsorbed Cr with respective percentages of elements and their Cr/Fe, Cr/Mn, and Cr/Sn are 29%, 55% and 1.8%, respectively.

**Table 1:** Elemental constitution of Fe_2_O_3_-MnO_2_-SnO_2_ ternary oxide nanoadsorbent.

Elements	Weight%	Atom%	Weight%	Atom%	Cr/Fe (wt%)	Cr/Mn (wt%)	Cr/Sn (wt%)
Before adsorption			After Cr(VI) adsorption				
**Fe**	2.97	7.43	4.85	5.70	29	55	1.8
**Mn**	5.24	12.87	2.58	3.09			
**Sn**	3.04	16.16	80.17	44.35			
**O**	88.75	63.54	10.98	45.08			
**Cr**	-	-	1.41	1.78			

### Batch studies

#### Effect of pH

**Fig 4:**
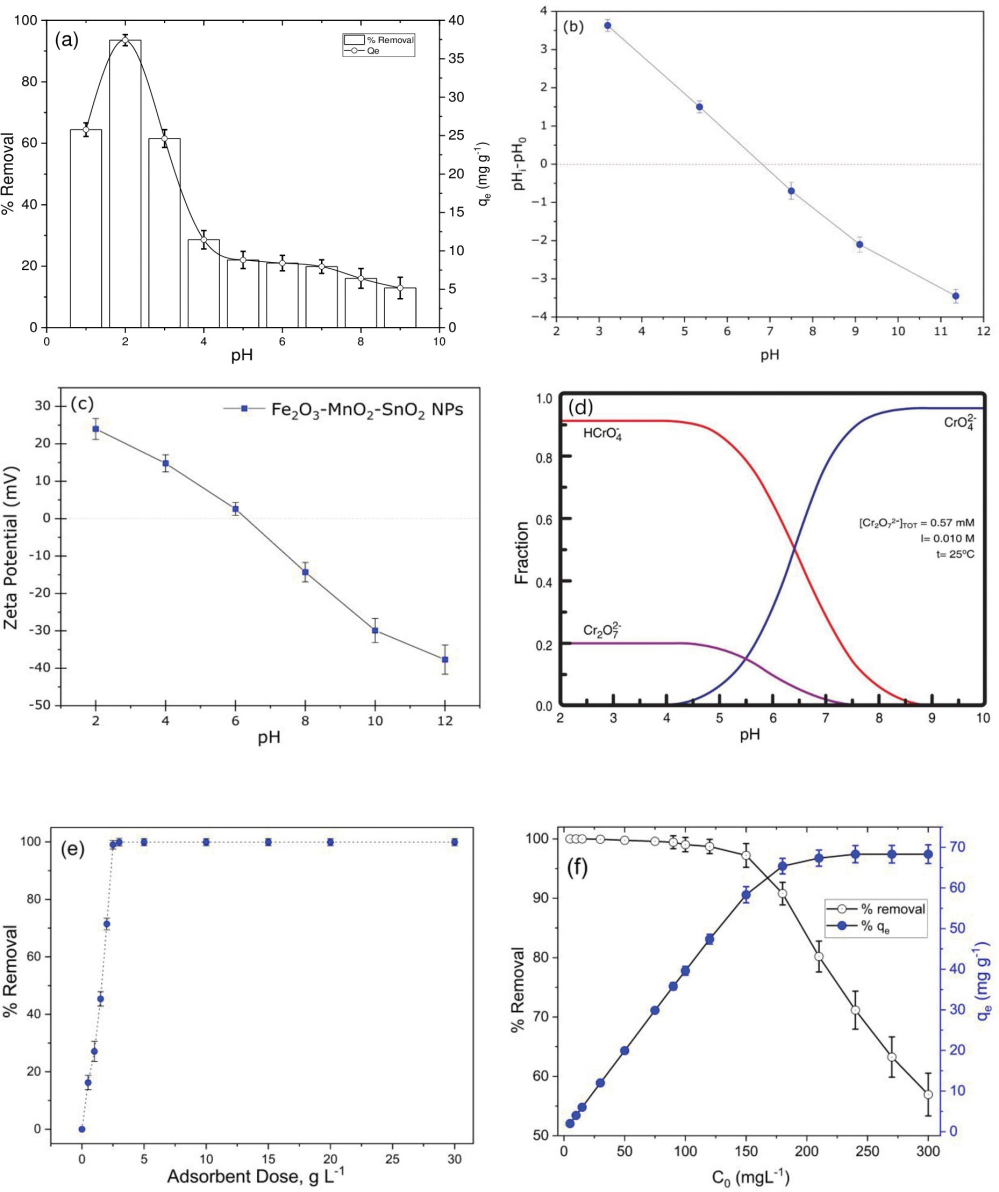
(a) effect of pH (pH 1–9, C_0_ = 100 mg L^-1^, adsorbent dose of 2.5 g L^-1^, agitation at 200 rpm, contact time 60 minutes, temperature 25°C), (b) determining pH_pzc_ of Fe_2_O_3_-MnO_2_-SnO_2_ NPs (c) distribution of zeta potential (d) Cr(VI) species in different pH (e) effect of Fe_2_O_3_-MnO_2_-SnO_2_ dosage (pH 2, C_0_ = 100 mg L^-1^, adsorbent dose of 0.5–30 g L^-1^, agitation at 200 rpm, contact time 90 minutes, temperature 25°C), (f) effect of initial Cr(VI) concentration (pH 2, C_0_ = 5.0–300 mg L^-1^, adsorbent dose of 2.5 g L^-1^, agitation at 200rpm, contact time 90 minutes, temperature 25°C).

The solution’s pH is a substantial part of adsorption studies because it significantly affects the adsorbent’s ability to remove metals from an aqueous medium. Fig 4(A) shows that the Fe_2_O_3_-MnO_2_-SnO_2_’s Cr(VI) adsorption intensely relies on pH. The solution’s initial pH influences the protonation and the adsorbent’s surface charge, in addition to the species of Cr(VI) in the mix. The absorption of Cr(VI) as a consequence of hydrogen ion intensity was investigated throughout a pH range of 1–9 and is illustrated in Fig 4(A) and it clearly exhibits that the adsorption characteristics of the adsorbents are strongly pH dependent. The observed outcomes discerns that the effectiveness of the produced adsorbents for adsorbing the Cr(VI) ion reduced as the pH increased. As noticed in Fig 4(A), when pH rises from 1 to 2, the efficiency of Cr(VI) removal improves from 79.4 to 93.6%, while adsorption capability improves from 25.77 to 37.42 mg g^-1^. Nonetheless, the Cr(VI) removal efficacy declines from 93.6 to 12.94% once the pH further rises from 3 to 9. Similar trend also found in the various studies [50]. Cr(VI) ion adsorption effectiveness reduced at increasing pH level; this decline may have been caused by many factors. Under various conditions, the primary chemical species of hexavalent chromium (Cr(VI)) present in a solution are Cr_2_O_7_^2^-, HCrO_4_−, CrO_4_^2^−, H_2_CrO_4_. The hydrolysis equilibrium reactions between these species are as follows:

$${Cr}_{2}O_{7}^{2-}+H_{2}O↔{2CrO}_{4}^{2-}+{2H}^{+}$$

$${Cr}_{2}O_{4}^{2-}+H^{+}↔{HCrO}_{4-}$$

$${HCrO}_{4-}+H^{+}↔{H_{2}CrO}_{4}$$

The reason for the good adsorption effect under acidic conditions is due to the existence of multiple O containing groups like hydroxyl, ester, and carboxyl groups on the adsorbent surface and form hydrogen bonds with HCrO4− in the solution. In contrast, the adsorbent’s surface is characterized by a substantial presence of Fe–OH groups, which facilitate the binding of H+ ions from the surrounding medium. As a result, the surface of the particles acquires a positive charge and undergoes protonation. Consequently, an electrostatic interaction occurs between the positively charged surface and the chromium-containing anions present in the aqueous solution. Furthermore, increasing the pH value inhibits Cr(VI) ion adsorption, resulting in a shift in adsorbents’ surface charges (from a positive charge to a negative one), which creates some repulsive electrostatic contact between the ions and the adsorbent surface [51]. Extensive research on the adsorption of Cr(VI) with the designated adsorbent was done, maintaining pH of the solution at 2.

*Zeta potential*. Both the surface charge and the degree of protonation of an adsorbent are profoundly affected by the solution pH. Zeta potential of the synthesized Fe_2_O_3_-MnO_2_-SnO_2_ nanoadsorbent was also examined at different pH values ranging from 2 to 12 to gain insight of the surface charge and Zeta potential of Fe_2_O_3_-MnO_2_-SnO_2_ is evident in Fig 4(B). The results shows that Fe_2_O_3_-MnO_2_-SnO_2_ NPs has a positive surface charge in the range of pH 1–6, which is incredibly favorable for Cr(VI) removal since there is an electrovalent bond between the nanoparticles and the chromium oxyanions. The zeta potential of Fe_2_O_3_-MnO_2_-SnO_2_ NPs declined with the increasing pH. The enhanced potential of H+ ions in solution is responsible for the greater zeta potential at low pH [52]. Zeta potentials, however, went negative in alkaline solutions because of the risen of OH^-^ ions.

It is observed from Fig 4D, that the predominant ion species for Cr(VI) at acidic pH is HCrO_4_^-^, while at basic pH, CrO_4_^-^ is the dominant species. The potential existence of anionic species at a lower pH allows for the formulation of a hypothesis suggesting that the removal of hexavalent chromium from the solution in Fe_2_O_3_-MnO_2_-SnO_2_ can be attributed to electrostatic phenomena. This hypothesis is supported by the fact that the pH at which the surface charge of the material is zero (pHpzc) is 6.7. Nevertheless, the adsorbent’s surface charge exhibited an increased negativity with the elevation of the solution’s pH. The presence of hexavalent chromium anion species predominates at alkaline pH levels, resulting in a decrease in the adsorption of Cr(VI). The aforementioned observation indicates that the primary mechanism for adsorption of hexavalent chromium from the aqueous phase using Fe_2_O_3_-MnO_2_-SnO_2_ is likely the electrostatic adsorption process.

#### Effect of Fe_2_O_3_-MnO_2_-SnO_2_ dosage

Fig 4(C) demonstrates how Cr(VI) adsorption efficiency increases with the Fe_2_O_3_-MnO_2_-SnO_2_ dosage, as a higher dosage means many more adsorption sites are available to the Cr(VI) ions [53]. This increase, however, only occurs up to a certain mass of adsorbent (2.5-g L^-1^), beyond which point there is no additional exponential improvement in adsorption. This means that the system has reached equilibrium at 2.5 g L^-1^ dosage (percentage of Cr(VI) adsorption = 98% is also highest at that point)—this was the value used as optimal dosage for the rest of the batch studies.

#### Effect of initial Cr(VI) ion concentration

The initial metal concentration is a crucial parameter influencing heavy metals adsorption from aqueous solutions. Initial adsorbate intensity in the liquid phase enhances the mass transfer rate between the solid and aqueous stages [54]. Fig 4(D) demonstrates the effect of the initial Cr(VI) intensity on adsorption onto the Fe_2_O_3_-MnO_2_-SnO_2_. The removal efficiency declines significantly after 150 mg Cr(VI) L^-1^ for Fe_2_O_3_-MnO_2_-SnO_2_, while its adsorption capacity increases as Cr(VI) ion concentration increases. This can be explained by the fact that while there is a constant total number of adsorption sites for a given fixed mass of adsorbent, the concentration of metal in the liquid phase fluctuates, lowering the removal effectiveness after equilibrium.

#### Adsorption isotherm

**Fig 5:**
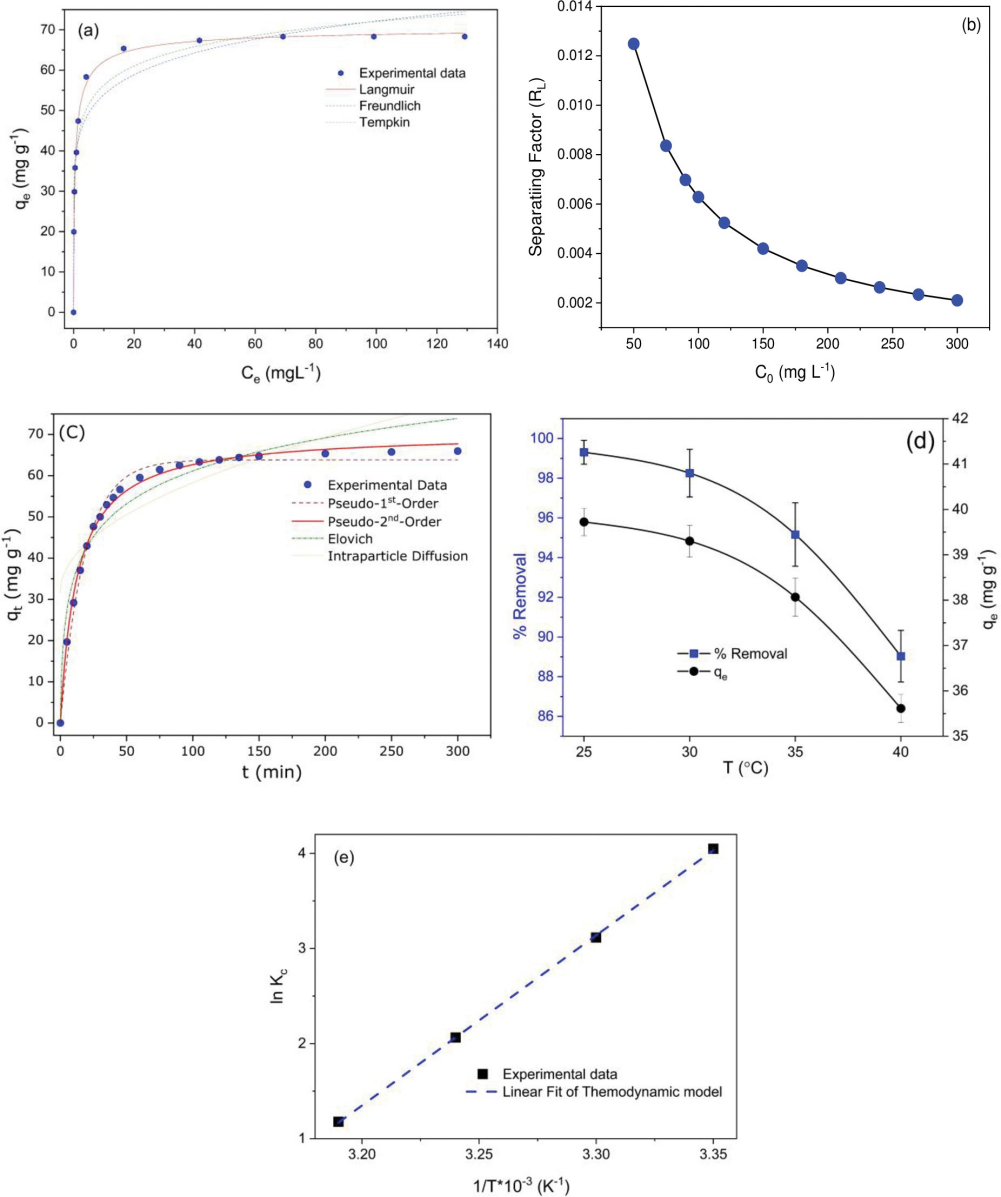
(a) Langmuir and Freundlich adsorption isotherm (pH 2, C_0_ = 50 mg L^-1^, adsorbent dose of 2.5 g L^-1^, agitation at 200 rpm, contact time 90 minutes, temperature 25°C), (b) the separation factor of Cr(VI) ions adsorption (c) Adsorption kinetics (pH 2, C_0_ = 180 mg L^-1^, adsorbent dose of 2.5 g L^-1^, agitation at 200 rpm, contact time 0−300 minutes, temperature 25°C), (d) effect of temperature (pH 2, C_0_ = 100 mg L^-1^, adsorbent dose of 2.5 g L^-1^, agitation at 200 rpm, contact time 90 minutes, temperature 25–40°C). (e) Van’t Hoff plot for the adsorption of Cr(VI) ions onto Fe_2_O_3_-MnO_2_-SnO_2_ NPs.

Fig 5(A) shows the non-linear fitted plot of the Langmuir Freundlich and Temkin isotherm models, while the isotherm data for Cr(VI) are shown in Table 2. The outcomes specify that the Langmuir model best fits the experimental data best (R^2^ = 0.999) over the Freundlich and Temkin model data. This Langmuir fit indicates Cr(VI) adsorption onto the surface of the Fe_2_O_3_-MnO_2_-SnO_2_ and maximum adsorption capacity qm obtained 69.2 mg Cr(VI) g^-1^, which was very close to its estimated adsorption capacity (qe) using the Langmuir isothermal model. The Langmuir model suggests that the adsorption of Cr(VI) is more probable on a homogeneous surface through monolayer adsorption, and no discernible interaction was observed between the adsorption targets [55]. A higher χ2 value also showing the suitability of the model for explaining the adsorption process for the studied systems. The separation (R_L_) factors for the various initial concentrations are illustrated in Fig 5(B) and summarized in Table 2. From the results, we find the following: 0 < R_L_ < 1, which points toward that the adsorption of Cr(VI) onto Fe_2_O_3_-MnO_2_-SnO_2_ was favorable. The investigation involved examining the potential for multilayer adsorption, taking into account the heterogeneous surface of the developed nanoadsorbent. This was accomplished by applying a non-linear regression analysis to the equilibrium adsorption data using the Freundlich adsorption isotherm. The model demonstrated a strong fit with the R^2^ and χ^2^value (Table 2), which was lower than that of the Langmuir model. This suggests that the model is not suitable for accurately describing the adsorption behavior. The Temkin adsorption isotherm model, which takes into account adsorbent-adsorbate interactions, was also fitted to the equilibrium adsorption data. The Temkin constant, B_T_, which is a measure of the heat of adsorption, was calculated by fitting the adsorption data to the Temkin isotherm model and values less than 8 kJ mol^-1^, favored the physisorption of metal ions onto the Fe_2_O_3_-MnO_2_-SnO_2_ nanoadsorbent.

**Table 2:** Isotherm parameters for adsorption of Cr(VI) onto Fe_2_O_3_-MnO_2_-SnO_2_ nanoadsorbent.

Adsorption Isotherms	Parameters	
Langmuir	q_m_	69.187 mg g^-1^
	K_L_	1.5819 L mg^-1^
	R_L_	0.0021–0.0125
	R^2^	0.999
	χ^2^	1.61
Freundlich	K_F_	40.28 (mg g^-1^).(L^-1^ mg^-1^)^1/n^
	1/n	0.0418
	R^2^	0.931
	χ^2^	38.92
Temkin	B_T_	6.868 J mol^-1^
	K_T_	8.320 L g^-1^
	R^2^	0.964
	χ^2^	20.07

#### Adsorption kinetics

The adsorption phenomena of gaseous molecules on the surfaces of porous materials are subject to the influence of surface heterogeneity, interconnected porosities, and the specific microporous or mesoporous structure of the adsorbent [56]. Models of pseudo-1^st^-order, 2^nd^-order, intraparticle diffusion, and Elovich kinetics models were exercised to fit the experimental data, as represented in Fig 5(C). The parameters of the kinetic models and coefficients attained from the non-linear fit are presented in Table 3.

**Table 3:** Kinetics parameters for adsorption of Cr(VI) onto Fe_2_O_3_-MnO_2_-SnO_2_ nanosorbent.

Adsorption Kinetic Models	Parameters	
Experimental	q_e_	66.07 mg g^-1^
Pseudo-first-order kinetics	q_e_	63.85 mg g^-1^
	k_1_	0.054
	R^2^	0.987
	χ^2^	3.208
Pseudo-second-order kinetics	q_e_	67.05 mg g^-1^
	k_2_	4.669
	R^2^	0.999
	χ^2^	1.073
Elovich	α	22.334 mg g^-1^ min^-1^
	β	0.086 g mg^-1^
	R^2^	0.95
	χ^2^	15.82
Intraparticle diffusion	k_id_	2.658 mg g^-1^ min^-1/2^
	C	31.74
	R^2^	0.91
	χ^2^	11.577

The experimental analysis reveals a more favorable correlation between the experimental data and the pseudo-second-order kinetics model, as evidenced by the highest coefficients of determination (R^2^) and the lowest values of the reduced chi-square (χ^2^). The first-order model assumes that solute uptake is directly proportional to saturation concentration and solid uptake over time, demonstrating physical adsorption [57]. However, pseudo-second order model assumes chemical sorption including valence forces and electron sharing between adsorbent and adsorbate [58]. The strong concurrence observed between the experimental and calculated qe values provides additional evidence in favor of pseudo-2^nd^-order kinetics. Numerous studies have documented that the adsorption mechanism of Cr(VI) onto nanoadsorbents follows second-order kinetics [59].

**Fig 6:**
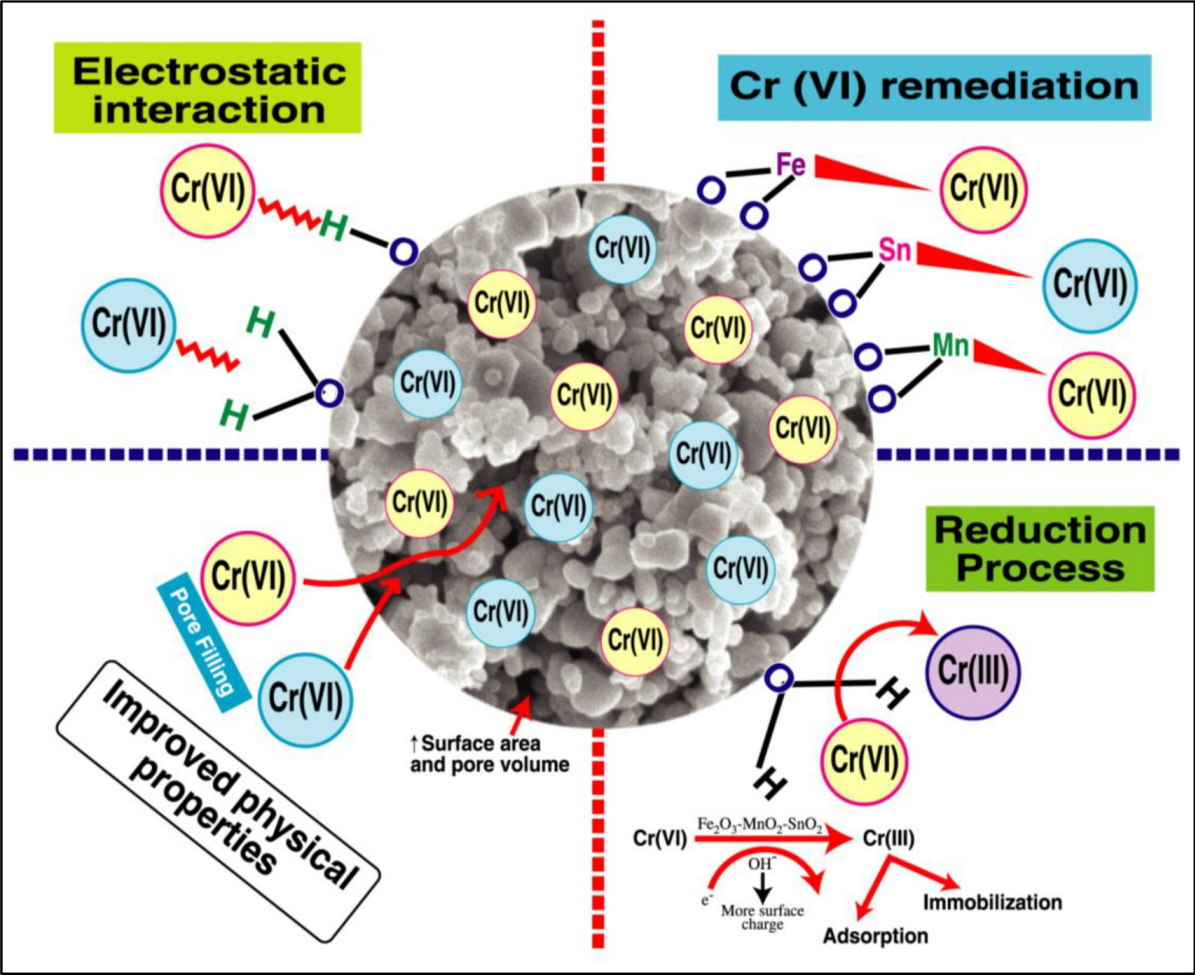
Proposed Cr(VI) mechanism by Fe_2_O_3_-MnO_2_-SnO_2_ NPs.

The adsorption process, as described in the literature, reportedly entails the diffusion of the adsorbate towards the adsorbent surface, where the adsorbate molecules compete with one another for adsorption onto the porous structure of the adsorbent [56]. The Elovich model’s usefulness, in addition to the inter-particle diffusion model, was thus explored. The initial adsorption rate calculated using the Elovich model was 22.332 g mg^-1^ min^-1^ with R^2^ of 0.95. The presence of a high rate constant for inter-particle diffusion and a significant inter-particle diffusion model constant (mg g^-1^) suggests the potential occurrence of a boundary layer effect [60]. Elovich and the inter-particle diffusion model are unfavorable for the systems under investigation because of their comparatively high values of χ^2^. The experimental results presented above provide evidence for a plausible mechanism of Cr(VI) removal by Fe_2_O_3_-MnO_2_-SnO_2_, as depicted in Fig 6.

#### Influence of temperature and thermodynamic analysis

Temperature may influence an adsorption system, thus, the effect of temperature on Cr(VI) adsorption was studied under varying temperatures: 25°C—40°C as shown in Fig 5(D). The Fig 4(E) reveals that the Cr(VI) adsorption declines from 39.7 mg g^−1^ to 35.6 mg g^−1^ with temperature increasing from 25°C to 40°C, indicating that the adsorption course of action is exothermic [51].

Besides this, thermodynamic constraints such as ΔH°, ΔS°, and ΔG° have been analyzed because they are quite crucial for deciding the mechanism of adsorption and its spontaneity. The expressions ΔS° and ΔH° are estimated as the intercept and slope of the *lnK*c against to the 1⁄*T* plot shown in Fig 5(E) and the significances are stated in Table 4. The negative values of ΔG° at different temperatures evidently confirm that the Cr(VI) adsorption is spontaneous and feasible [61], while ΔH°’s negative value proves the way of adsorption is exothermic. Besides, the findings demonstrate that the sorption process is exothermic, as evidenced by the decreased movement of metal ions at higher temperatures. Likewise, the positive value of ΔS° suggests that the interaction of Cr(VI) over solid/liquid interfaces is random, and the mechanism of adsorption is entropy-dominated [62]. Additionally, the value of ΔH° and ΔG° illustrates how Cr(VI) ions interact with Fe_2_O_3_-MnO_2_-SnO_2_ i.e., whether it is was physisorption or chemisorption. Physisorption is typically observed when the ΔG° value falls within the range of -20 to 0 kJ mol^-1^, whereas chemisorption is observed when the ΔG° value falls within the range of -400 to -80 kJ mol^-1^ [63]. The determination of ΔH° values provides a means to investigate the adsorption mechanism. When the value of ΔH° is below 29 kJ mol^-1^, it is generally classified as physisorption. On the other hand, ΔH° >29 kJ mol^-1^ is indicative of chemisorption bonds [64]. The value ΔG°and ΔH° agrees that the adsorption process is physisorption. The adsorption entropy change ΔS° greater than zero favored the adsorption by Fe_2_O_3_-MnO_2_-SnO_2_ nanoadsorbent.

**Table 4:** Thermodynamic parameters for Cr(VI) removal onto Fe_2_O_3_-MnO_2_-SnO_2_ nanosorbent.

T(K)	C_e_	q_e_	K_c_	ΔG° (KJ mol^-1^)	ΔH° (KJ mol^-1^)	ΔS° (J mol^-1^ K^1^)
298	0.69	39.72	57.57	-9.20		
303	1.74	39.30	22.59	-7.08	-0.44	140.10
308	4.83	38.07	7.88	-4.69		
313	10.96	35.62	3.25	-2.67		

#### Desorption and regeneration study

**Fig 7:**
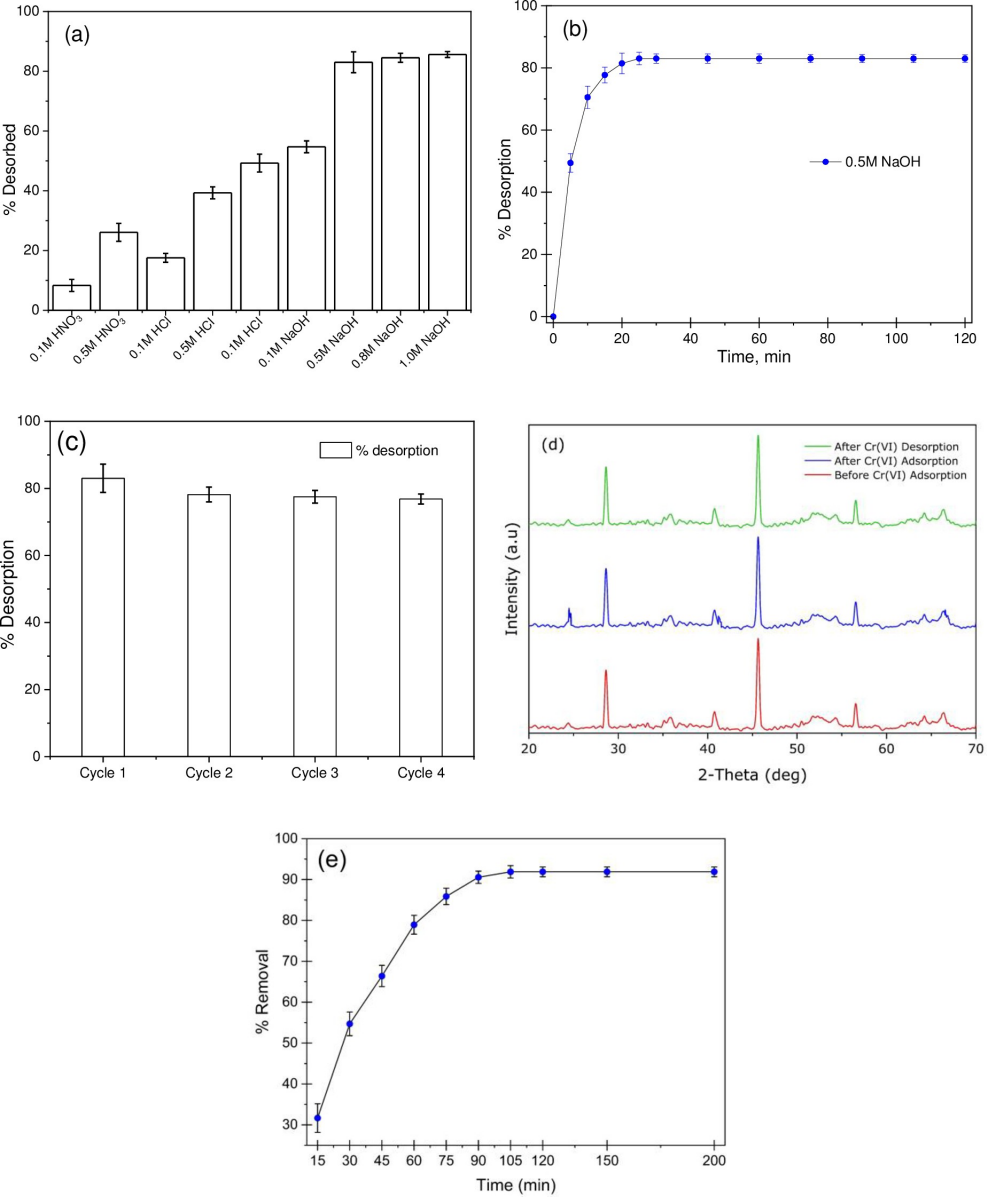
Desorption and regeneration efficiencies of Fe_2_O_3_-MnO_2_-SnO_2_ nanoparticles (a) influence of desorbing agents on Cr(VI) desorption potential, (b) influence of desorbing time on Cr(VI) ions using 0.5M NaOH, (c) regeneration stability experiment of Fe_2_O_3_-MnO_2_-SnO_2_ nanoparticles performed using a 100 mg L^-1^ of Cr(VI), 200 rpm agitation speed, 90 minutes removal time, 0.5M NaOH desorbing reagent, and 120 minutes desorbing time. (d) XRD patterns before adsorption, after adsorption and desorption of Cr(VI) (e) Adsorption of chromium from tannery wastewater in optimum dose and conditions as per batch experiment.

Studying the regeneration properties of an adsorbent is crucial because its reusability determines treatment costs [NO_PRINTED_FORM]. Thus, the study of desorption is vital for understanding whether the adsorbent material is economically viable or not. In this study, the desorption efficacy achieved is shown in Fig 7(A), where a low desorption efficiency has been observed when using HCl (0.10 M, 0.50 M, 1.0 M) and HNO_3_ (0.10 M, 0.50 M) as the eluent. However, the NaOH solution (from 0.5, 0.8, and 1.0 M) shows significant performance in desorbing Cr(VI) ions from the nanoadsorbent. This means that a basic eluent is more preferable than an acidic eluent to desorb the Cr(VI). Since the presence of hydroxyl groups in a basic eluent competes with the negatively charged Cr(VI) ions for binding sites during desorption, Cr anions get replaced by OH^-^ groups on the Fe_2_O_3_-MnO_2_-SnO_2_ surface. Fig 7(B) illustrates the desorption rate of Cr(VI) ions using 0.5-M NaOH eluent. Four adsorption-desorption cycles have been completed to study the reusability of the Fe_2_O_3_-MnO_2_-SnO_2_. Fig 7(C) shows that the desorption efficiency is found to be consistent (~80%) over the four cycles. The loss of adsorption capacity (about 23% after the four cycles) can be associated with the development of metal complexes on the surface of the Fe_2_O_3_-MnO_2_-SnO_2_ nanoadsorbent. To assess the stability of the developed nanoadsorbent, XRD test has been done in three events, (before and after adsorption: after desorption of Cr(VI). The result is depicted in Fig 7(D) which represents that during these three events no significant change has been observed. Furthermore, no Fe_2_O_3_-MnO_2_-SnO_2_ residue has been traced during the leaching test, which indicates the developed nanoadsorbent doesn’t degrade promptly.

The results of a comparison between the adsorption capacity of Cr(VI) achieved using the Fe_2_O_3_-MnO_2_-SnO_2_ nanoadsorbent and that of other ternary materials described in the literature are presented in Table 5. It is clear that the Fe_2_O_3_-MnO_2_-SnO_2_ nanoadsorbent has a high adsorption capacity in comparison to other adsorbents. Moreover, ternary materials of Fe_2_O_3_-MnO_2_-SnO_2_ are significantly stronger to binary or other ternary ones in their ability to remove chromate ions.

**Table 5:** Adsorption comparisons between different adsorbates and adsorbents.

Nano Adsorbent for Cr(VI) removal	Preparation methods, reaction time	S_BET_ m^2^ g^-1^	pH	Cr(VI) concentration (mg L^-1^)	m_ads_ (mg L^-1^)	q_m_ (mg g^-1^)	Fitted Isotherm	R^2^	Ref
**This work**	Sol-gel, 1.5h	14.539	2	100	25	69.2	Langmuir	0.999	
Fe_3_O_4_-FeB	Sol-gel	15.4	6.3	32	0.8	38.9	Langmuir	0.99	[65]
GO-Fe_3_O_4_	Pyrolysis	-	2	25	40	3.197	Langmuir	0.998	[66]
Magnetic magnetite (Fe_3_O_4_)	-	50	4	-	2	8.67	Langmuir	0.975	[67]
CuFeCr-LDH	Microwave hydrothermal, 2 h	100.3	2	25	50	22.24	Langmuir	0.998	[68]
α-Fe_2_O_3_@C	Decomposition under thermal condition, 6h	-	3	25	-	76.92	Langmuir	0.99	[69]
CH–Mg/Al/Fe	2 h	-	3	50	100	52.5	Langmuir	0.999	[70]

#### Adsorption of chromium from tannery wastewater

TCT Ltd. at the BSCIC Tannery Industrial Area in Hemayetpur, Savar, Dhaka, Bangladesh, was the source of the wastewater which was used in the experiment. Sufficient precautions were taken while sampling to ensure that no extraneous pollutants would affect the outcomes. Table 6 contains information on the characteristics of the collected tannery’s effluent. The samples were filtered using 0.45 microns’ filter paper and then it was adjusted to 100mL with de-ionized water. The sample was then analyzed using AAS to know the concentration of chromium in the sample. Then the effectiveness of the Fe_2_O_3_-MnO_2_-SnO_2_ nanoadsorbent was investigated to know the efficacy in Chromium removal from the real wastewater. The optimal adsorbent dosage, sample pH, and other experimental parameters were used in this process. In the wastewater initial chromium concentration was 87.39 mg L^-1^ with other metals i.e., copper (0.4294 mg L^-1^), lead (0.172 mg L^-1^). Fig 6(E) demonstrates that before equilibrium was reached, there was a quickening in the rate at which chromium ions were removed from the effluent. It was discerned that increasing the contact period from 15 to 90 minutes improved Cr(VI) elimination from 31.66% to 90.55%. After 105 minutes, the Cr(VI) removal rate is still stable at 91.89%, indicating that equilibrium was reached at that point. Adsorbent efficiency was found to be low, even though the concentration of chromium in the wastewater was lower than that employed in the laboratory experiment due to the presence of trace amounts of lead and copper along with other contaminations.

**Table 6:** Characteristics of tannery wastewater collected from the CTL, Savar.

Parameters	Unit	Wastewater
pH		9.2
DO	mg L^-1^	2.62
TDS	mg L^-1^	21620
TSS	mg L^-1^	1690
EC	μS cm^-1^	5927
BOD_5_	mg L^-1^	5464
COD	mg L^-1^	16840
Pb	mg L^-1^	0.172
Cu	mg L^-1^	0.4294
Total Cr	mg L^-1^	87.39
Temperature	°C	35
Cl^-^	mg L^-1^	12.65

## Conclusions

For the first time, to remove Cr(VI) ions from an aqueous solution, a novel ternary oxide adsorbent (Fe_2_O_3_-MnO_2_-SnO_2_) was developed using the sol-gel technique. Structure features, adsorption properties, and the mechanism of removal of Cr(VI) from an aqueous solution were investigated to determine the effect of Fe_2_O_3_-MnO_2_-SnO_2_ on Cr(VI) removal. Based on the experimental findings, we have come to the following conclusions:

- XRD, FTIR, N_2_ adsorption-desorption, and TGA were all successfully used to characterize the produced nanoadsorbent. The synthesis of the pure ternary nanoadsorbent is confirmed by the presence of peaks in the FTIR data corresponding to the various functional groups in Fe_2_O_3_, MnO_2_, and SnO_2_. Besides, the XRD results show that the average size of a single crystalline particle is 39.27 nm, with a range of 23.52 nm to 49.99 nm. In addition, BET surface has been found to be 14.539 m^2^ g^-1^, where the mean pore diameter of the nanoparticle was calculated to be 4.96 nm. This value demonstrates that mesoporous nanoparticle production has taken place. Congruently, the EDX analysis confirms that Fe_2_O_3_-MnO_2_-SnO_2_ has the same basic components everywhere over its surface: Fe, Mn, Sn, and O.
- The adsorption of Cr(VI) onto Fe_2_O_3_-MnO_2_-SnO_2_ was greatly pH-dependent, and the optimum pH was 2.0.
- The ternary Fe_2_O_3_-MnO_2_-SnO_2_ adsorbent demonstrated rapid kinetics: equilibrium was achieved within 90 minutes with a maximum adsorption capacity of 69.2 mg Cr(VI) g^-1^.
- The adsorption process obeyed pseudo-second-order kinetic model and the adsorption isotherm suited nicely with the Langmuir isotherm.
- Thermodynamic studies show that the Cr(VI) ion adsorption onto Fe_2_O_3_-MnO_2_-SnO_2_ was an exothermic and spontaneous process.
- Although the regeneration efficiency was found to be consistently about 80% using 0.5-M NaOH over four cycles of adsorption-desorption, around a 23% fall in adsorption capacity was observed, perhaps as a result of metal complexes forming on the Fe_2_O_3_-MnO_2_-SnO_2_’s surface.
- Adsorption of chromium from the real wastewater shows good efficiency of Fe_2_O_3_-MnO_2_-SnO_2_ as adsorbent
